# Hypertrophic cardiomyopathy: from mutation to functional analysis of defective protein

**DOI:** 10.3325/cmj.2011.52.384

**Published:** 2011-06

**Authors:** Pavel Capek, Jiri Vondrasek, Jiri Skvor, Radim Brdicka

**Affiliations:** 1 Department of Anthropology and Human Genetics, Charles University, Prague, Czech Republic; 2 Institute of Organic Chemistry and Biochemistry – Center for Complex Molecular Systems and Biomolecules, Prague, Czech Republic; 3 Institute of Biophysics and Informatics, First Faculty of Medicine, Charles University, Prague, Czech Republic; 4 Department of Molecular Genetics, Institute of Hematology and Blood Transfusion, Prague, Czech Republic

## Abstract

**Aim:**

To analyze the genesis of hypertrophic cardiomyopathy on a large cohort of patients from molecular genetics point of view and perform the functional analysis of the 3D molecular model of defective myosin-7 protein *in silico*.

**Methods:**

The study enrolled 153 patients with diagnosed hypertrophic cardiomyopathy from different parts of the Czech Republic. DNA samples were analyzed for mutations in exons 21 and 22 of the *MYH7* gene, which have been associated with high mutation clustering. The 3D model of human myosin-7 was built using the x-ray structure of nucleotide-free scallop myosin S1 as the structural template. We performed *de novo* structure prediction of mutant and wild type peptides spanning the 769-788 amino acids region of the myosin-7 protein.

**Results:**

The Arg^870^His and Asp^778^Val amino acid alterations were found in 2 unrelated patients with a severe form of hypertrophic cardiomyopathy. The Asp^778^Val variation was chosen for subsequent 3D molecular modeling *in silico*. The mutation of the Asp by Val not only changes the character of the interaction pattern with other amino acids or ions but Val, being a small hydrophobic amino acid, can also completely change the stability of the region.

**Conclusion:**

Mutation location in the *MYH7* gene and changes in amino acid composition may have a crucial negative impact on the outcome of the disease in patients with hypertrophic cardiomyopathy. In addition, a mutation that changes the charge of the amino acid is more likely to affect protein function than a conservative mutation.

Hypertrophic cardiomyopathy (HCM) is a complex inheritable cardiac disease that is highly clinically and genetically heterogeneous. Leading macroscopical clinical features of HCM are left and/or right ventricular hypertrophy, which in most cases is asymmetric with the involvement of the intraventricular septum in absence of other causes of hypertrophy (eg, valvar stenosis and hypertension). However, the symmetrical form of HCM accounts for over one third of cases and is characterized by concentric thickening of the left ventricle with a small ventricular cavity dimension (1). The prevalence of the HCM in the general population is 0.2% according to the echocardiographic criteria (2). From a genetic point of view, HCM is a congenital cardiac disease with autosomal dominant pattern of inheritance and incomplete penetrance (3). In some families, the onset of disease occurs late in adulthood, the hypertrophy is minimal, and patients have a normal lifespan, while in other families the onset occurs very early and may lead to a massive hypertrophy associated with severe symptoms and a very short lifespan due to a sudden cardiac death (4-7). There are many risk factors linked with the worsening of HCM, such as diabetes mellitus, high levels of cholesterol, and a number of prothrombotic abnormalities. Therefore, long term attention should be paid to the testing of new drugs that reduce the production of inflammatory aggregates/plaques. The correction of these aberrations may translate into the reduction of cardiovascular risk in patients with HCM (8,9).

HCM is mostly attributed to multiple mutations in approximately 16 genes that have been identified until now (10-12). Mutations associated with HCM development have been found in genes that encode components of the thick filament proteins: myosin-7 and myosin-binding protein C and in genes that encode the components of the thin filament proteins: cardiac troponin T (13), cardiac troponin C, cardiac troponin I, and α-tropomyosin (14,15). *MYH7*, a gene encoding myosin-7 protein, was the first gene to be linked with HCM development (16). It is a large gene located on the chromosome 14q11.2–q13 in humans with 40 exons forming a transcript of 6027 bp. The size of myosin-7 is 1935 amino acids. The head region extends from exon 3 to part way through exon 21, the neck from part way through exon 21 to part way through exon 25, and the tail from exon 25 to 40. The functional sites of myosin-7 are as follows: 1) ATP-binding domain (exons 5-12), 2) actin-binding domain (exons 13-16), and 3) light chain-binding sites extends through exon 21 and 22 of the *MYH7* gene.

The genetic part of the study focused on the mutation screening of the exons 21 and 22 of the *MYH7* gene. The selected exons encode light chain-binding sites of the myosin-7 protein. High mutation clustering within this region was reported by many scientists (17). An identification of pathogenic mutation might be beneficial in the process of preclinical diagnosis and in genetic counseling. More mutations occur in the head and neck than in the tail of myosin-7. In the head region, 130 mutations have been reported in association with HCM development (size of the region: 778 amino acids), 52 in the neck (289 amino acids), and 25 in the tail (868 amino acids). It is widely agreed that mutations in the head region of the *MYH7* gene are more likely to lead to a severe form of HCM (18).

The diagnosis of HCM depends on the molecular identification and analysis of the candidate genes and of the abnormal gene product (3,19). To be able to understand how mutations in different genes, especially those that encode for contractile proteins cause HCM, it would be necessary to understand the functional consequences of the mutations at the molecular level (11). With the knowledge of the responsible genes and the ability to detect the underlying genetic and proteomic defect, we will be able to determine whether specific genotypes lead to different phenotypes (20-22).

The aim of this study was to analyze the HCM genesis from the molecular genetics point of view, perform a functional analysis of defective myosin-7 protein *in silico,* and investigate specific mutations that change the charge of the amino acids responsible for the HCM development.

## Material and methods

### Molecular genetic analysis of exons 21 and 22 of MYH7 gene

A total of 153 patients from different parts of the Czech Republic were enrolled to this study. All of the collected samples were fully anonymous and were given voluntary during the period 2000-2003. Informed consent was obtained from all study participants. The DNA bank of the Department of Anthropology and Human Genetics, Faculty of Science, Charles University in Prague houses all the tested samples and all procedures were carried out in line with the institutional ethical guidelines. This cohort was divided into two major subgroups: 1) patients with a sporadic form of HCM in whom HCM had been clinically proven by echocardiography and no family history of HCM had been reported (n = 102), 2) patients with a familial HCM in whom positive HCM occurrence had been previously found in at least one of the family members – 24 families with a familial form of HCM diagnosis had been identified to meet this criterion (n = 51).

DNA was extracted from peripheral blood leukocytes by phenol-chloroform extraction. Conditions for the polymerase chain reaction (PCR) were as follows for exons 21/22: 1.5 mM MgCl_2_/ 2 mM MgCl_2;_ 10 × PCR Buffer (70 mM KCl, 14 mM Tris-HCl); 2 mM dNTPs; 0.20 U Taq (5U/µL) (TaKaRa Bio, Inc., Shiga, Japan) and primers (each of total 10 µM concentration). Total reaction volume was 10 µL and we used 1-10 ng of template DNA per reaction (quantified by spectrophotometry). PCR was performed for amplification of specific regions of the genomic DNA (PCR program: 30 cycles, 94°C/5 minutes, 60°C/30 seconds for exon 21, and 62°C/30 seconds for exon 22, 72°C/1 minute, final extension 72°C/10 minutes, and 4°C hold). Oligonucleotide primer sequences were as follows: for exon 21: 5′- TAG GCT GTT ACC CTT CCT AAG GTA - 3′; 5′- GCC TCT GAC CCT GTG ACT GCA GTG - 3′ and exon 22: 5′- GGA CCT CAG GTA GGA AGG AGG CAG - 3′; 5′- TGT GCA GGG AGG TGC AGG GTT GTG - 3′.

All tested samples were analyzed by automatic dye terminator cycle sequencing using genetic analyzer ABI Prism 310 (Applied Biosystems, Foster City, CA, USA). DNA samples were analyzed for mutations in the nucleotide sequence of exons 21 and 22 of the *MYH7* gene, which are associated with a high number of mutations.

### Homology model of human myosin-7

The 3D model of human myosin-7 was built using the x-ray structure of nucleotide-free scallop myosin S1 (protein data bank accession code 1KK8) as the structural template. Based on global pair-wise alignment of the human (Uniprot id P12883) and scallop myosin (Uniprot id P24733) performed by EMBOSS Pairwise Alignment Algorithms (The European Molecular Biology Open Software Suite, *http://www.ebi.ac.uk/Tools/emboss/align/*) with BLOSUM62 (blocks of amino acid substitution matrix), replacement of the structurally conserved regions and rebuilding of the said variable regions was done with the homology module of molecular operating environment program (Chemical Computing group Inc., Montreal, Canada). Root mean square deviation of the framework (C_α_) was about 2.4 Å in the energy optimized model of myosin-7 protein compared with scallop myosin. Only the region spanning amino acids 1 to 835 of the myosin-7 was modeled because mutation in question – Asp^778^Val was present in this part of the structure.

### Short peptides mapping 769-788 amino acids region of myosin-7

We performed a *de novo *structure prediction of mutant and wild type peptides spanning the 769-788 amino acids region of myosin-7. The first peptide sequence contained the Asp amino acid at position 778 – LLGLLEEMR**D**ERLSRIITRI, while the second peptide was Asp^778^Val mutant variant of the wild type myosin-7 fragment – LLGLLEEMR**V**ERLSRIITRI. We used the web based prediction PEP-FOLD server (peptide structure prediction server, *http://bioserv.rpbs.univ-paris-diderot.fr/PEP-FOLD/*) to obtain 3D models of the peptides (wild type and mutant variant). PEP-FOLD method is based on structural alphabet and uses a greedy algorithm and a coarse-grained force field to predict a structure (23).

## Results

Two sequence alterations were found in 2 unrelated patients with the severe form of HCM. These 2 patients belong to the first subgroup of patients with the sporadic form of HCM in which no family history of the disease and sudden cardiac death was reported. The patient with the Arg^870^His substitution was 36 years old at the time of diagnosis with the left ventricular wall thickness of 28 mm and the left ventricular outflow tract gradient of 60 mm Hg. The patient with the Asp^778^Val mutation was 23 years old at the time of diagnosis with the left ventricular wall thickness of 32 mm and the left ventricular outflow tract gradient of 90 mm Hg. The Arg^870^His (codon change: C**G**C>C**A**C; nucleotide position in the *MYH7* gene: 14593) mutation was observed in the exon 22 of the *MYH7* gene. The Asp^778^Val (codon change: G**A**C>G**T**C; nucleotide position in the *MYH7* gene: 14061) was reported for the first time in a European patient with HCM. This mutation was detected in the encoding sequence of exon 21 of the *MYH7* gene (24). Both of the patients had chronic atrial fibrillation, chest pain, fatigue, and dyspnea. The patient with Asp^778^Val had had a few episodes of syncopes before the treatment. Drug treatment included the use of beta blockers and calcium channel blockers.

The Asp^778^Val amino acid alteration was chosen for subsequent molecular modeling, since we believe that it can play an important role in the molecule behavior.

### Homology model of the myosin-7 protein

**Figure 1:**
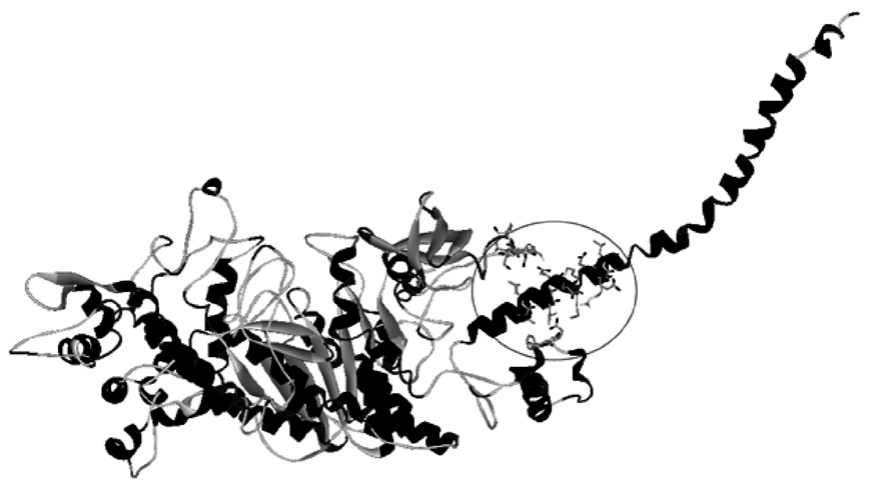
The homology model of myosin-7. Region of interest is highlighted.

A homology model of the myosin-7 N-terminal, motor domain, and 5 amino acids extended EF hand (the motif containing helix-loop-helix structural domain found in a large family of calcium-binding proteins) binding site (1-779 amino acids, 780-835 amino acids) was created using scallop myosin as a template (PDB 1KK8). The full length alignment of both full sequences of length 1958 amino acids showed relatively good agreement to build a homology-based model. The identity of both chains was 55.8% (1092/1958) and the similarity was 74.6% (1460/1958). The homology-modeled part of myosin-7 (1-835 amino acids) provided almost the same identity (61.2%) and similarity (71.2%). Two structures of myosin-7 were obtained representing two variants of the protein – the wild type and the Asp^778^Val mutant. There is no difference between these two models at general molecular level (Figure 1). Even the Asp^778^Val mutant shows the same helicity, spanning the 761-830 amino acid region.

The model shows that the aspartic acid at position 778 is located at the beginning of the long helix (starting Thr^761^) in charge-rich environment – Glu, Asp, Arg. One can assume that extensive solvation takes place in this region or that the region is important for its interaction dependent on Ca^2+^. Mutation of the Asp by Val not only changes the character of the interaction pattern with other amino acids or ions but Val, being a small hydrophobic amino acid, can completely change the stability of the region.

### Short peptides mapping the 769-788 amino acids region of myosin-7

**Figure 2:**
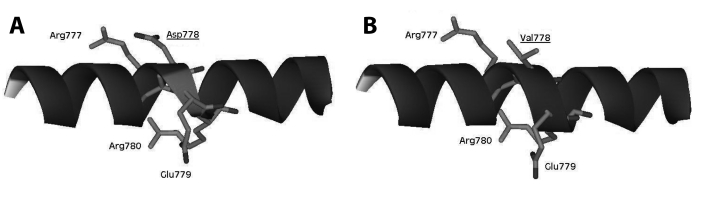
(**A**)The peptide spanning the region in which the mutation was located. Aspartic acid makes salt bridge with the arginin and therefore introduces a tension into the presented helix. (**B**) If Asp^778^Val mutation is present there is no deviation from the helicity during the simulation.

The core idea behind the model of short peptides mapping 769-788 amino acids region was to localize the differences in predicted peptide structures suggesting how the property of one amino acid can change the quality or the dynamics of the short sequence in question. As follows from the predicted structures, both regions are helical but there is one very important difference. The Asp^778^ is stabilized by interaction with Arg^777^, and Glu^779^ is stabilized by interaction with Arg^780^ (Figure 2A). This introduces a tension in the helix and indeed, the predicted structure shows measurable difference from an ideal helicity. Contrary to Asp, the Val in position 778 does not destabilize the structure of the helix and seems to be an important stabilizing element of this part of the structure (Figure 2B). We do not have any information about dynamical behavior of this part of myosin-7, but dynamical behavior of both peptides is quite different. Short molecular dynamics simulation (10 nanoseconds) in an explicit solvent revealed that rigidity of the Asp^778^Val mutant of the peptide was 5-6 times higher than of the Asp^778^ variant of the peptide.

## Discussion

We found Asp^778^Val alteration in exon 21 and the Arg^870^His in exon 22 of the *MYH7* gene. These exons are mainly mutated in cardiomyopathy patients and a presence of such a mutation is often considered to be linked with a very poor prognosis (25,26). Based on the results of the molecular genetic part of this study, a 3D model of myosin-7 was built. Structural prediction and functional analysis of mutant and wild type variant of myosin-7 fragments showed the increased rigidity of the Asp^778^Val mutant affecting the dynamics and proper function of the myosin-7 protein. We hypothesize that it can change the dynamics and flexibility of the long helical part or it can modify its interaction property.

The 3D model of human myosin-7 was built using the x-ray structure of nucleotide-free scallop myosin S1 (1KK8) as the structural template. There are other models of human myosin-7 in databases – 1KK2 in PDB database and the model at Protein Model Portal (*http://www.proteinmodelportal.org/query/uniprot/P12883*) based on the alignment of human myosin-7 with a sequence of *Gallus gallus* myosin, the structure of which was recently published (PDB ID 2MYS, sequence identity 79%). Structural alignment of our model with both obtained models showed similar root mean square deviation of about 14 Å. In both cases, the greatest part of the difference is caused by distortion of the C-terminal helix containing the studied mutations Asp^778^Val. Moreover, the part around residue 778 in 1KK2 model is significantly non helical, whereas both other models provide good agreement in this part.

All the patients from the cohort underwent the entry cardiological and clinical examination and this provided the information about the disease severity in HCM patients. However, long-term follow-up would be beneficial to monitor the development of the HCM in patients with mutations observed in this research.

In contrast to other studies, genetic results from this study show an unusually low number of mutations in the *MYH 7* gene in the tested regions (14,27-29). Only Roncarati et al have recently reported a low frequency of mutations in the *MYH 7* gene in a large cohort of Italian patients with HCM (30). This indicates that there is a need for a more inclusive investigative approach in order to fully understand the pathogenesis of this disease.

Mutation location in the *MYH7* gene and the resulting changes in amino acid composition may have a negative impact on the disease outcome in patients with HCM. Due to the different properties of the globular head domain (S1), neck or hinge domain (S2), and tail (light meromyosin) domain of the myosin-7 (31), mutations may have diverse effects depending on their location. In addition, as a mutation can give rise to a change in the amino acid sequence, the structure and interactive properties of the mutant protein may also be altered. Therefore, the positioning of the mutations along the gene and protein may offer insights into the mechanism by which normal protein function is impaired. It is hypothesized that a change in the amino acid charge may affect the severity of the phenotype (18).

The Arg^870^His substitution was first reported by Rayment et al. According to their research, defect of the Arg^870^His alteration lies in the transmission of force to the thick filament array. This could affect the assembly of the thick filament or stability of the protein. If such defective myosin-7 proteins are present at the same levels as the wild type, the effect may be due to a loss of tensile strength or rigidity in this part of the molecule (31). According to Cuda et al, myosin samples isolated from the soleus muscle tissue bearing the Arg^870^His mutation moved actin filaments more slowly than 22 control samples (32). It was reported that because both Arg and His residues are classified as basic amino acid residues, the nature of Arg^870^His mutation does not involve the change in charge (33). Nishi et al found that Arg^870^His mutation might not cause severe clinical manifestation in young persons (33). The possible defect of Arg^870^His is well documented and therefore we focused on the structure prediction and functional analysis of the 3D molecular model of myosin-7 (mutant “Asp^778^Val” and the wild type variant) *in silico*.

The Asp^778^Val alteration was first reported by Van Driest et al as a result of the comprehensive analysis of the *MYH7* gene in 389 unrelated patients with HCM in the USA (34). To our knowledge, no previous study performed 3D molecular modeling and a structure/function prediction of the wild type and mutant variant (Asp^778^Val) of myosin-7.

The mutation of the Asp by Val not only changes the character of the interaction pattern with other amino acids or ions but Val, being a small hydrophobic amino acid, can completely change the stability of the region. We hypothesize that it can change the dynamics and flexibility of the long helical part or it can modify its interaction property. The Asp^778^Val amino acid alteration is situated in a region that is highly conserved inside of the known sequence of the myosin-7 protein, indicating that such a mutation may have a crucial structural and functional impact. As follows from the homology model and from the modeled peptides, there are at least two principal aspects that can possibly alter functionality of this domain dramatically. The first is the intramolecular stabilization pattern dependent on Asp^778^ interaction with Arg^777^, which on the other hand can destabilize the helicity and make this part more dynamic. The second aspect is related to the dynamics of the Asp^778^ and Asp^778^Val, which are different suggesting that Asp is important for proper flexibility or probably the structure stability of myosin-7, and Val in this position increases rigidity, which seems to be counterproductive. Rigidity of the Asp^778^Val mutant can be very important factor in the dynamics of the myosin-7, together with other factors – Ca^2+^ affinity, EF hand-binding properties, and solvation of the region. This mutation may have negative impact on myosin, which generates less force and leads to the stimulus for compensatory hypertrophy (35).

There are other two mutations that have been reported in the codon 778: 1. Asp^778^Gly (36) and Asp^778^Glu (37). If we follow our hypothesis that the flexibility of the region is the feature that influences the function, then the Asp^778^Glu should produce similar effect as the Val or Gly. All the amino acids – Val, Asp, and Glu (with the Gly as the only exception) have similar propensities for helical arrangement. Therefore, one CH2 longer side chain of the Glu can create the salt bridge with the Arg^777^ without conformational stress, and therefore this mutation keeps the helix relatively untouched and similarly rigid as the Val or Gly.

In addition, a mutation that changes the charge of the amino acid is considered more likely to affect protein function than a conservative mutation as it was hypothesized by Ng and Henikoff (38). The role of charged residues can be divided into 4 major categories: a) creating a salt bridge bonds and stabilizing a structure; b) having a specific role in active sites of some enzymes; c) binding ions or metal atoms and coordinating their position in a structure; and d) increasing solubility of the protein and participating on protein-protein interactions. In the case of myosin-7, it is apparent that all categories except b) can take place. Synergy of all these effects could modify protein function significantly more than if applied separately.

In conclusion, we found two mutations in the exons 21 and 22 of the *MYH7* gene in two unrelated patients with severe form of HCM with no family history of HCM. The Asp^778^Val amino acid alteration was chosen for subsequent molecular modeling. For proper function of myosin-7, the Asp is important and Val in this position increases rigidity, which seems to be counterproductive. This amino acid change may lead to the decreased contractility of myosin molecule.

The 3D molecular models and structure predictions of defective proteins involved in the disease development followed by functional analysis *in silico* could be essential in research on HCM in the very near future.
